# Leukemoid Reaction With Severe Diabetic Ketoacidosis

**DOI:** 10.7759/cureus.50325

**Published:** 2023-12-11

**Authors:** Tharwat W Hussein, Zahra D Almualem, Fatema D Almualem, Bushra A Zainaldeen, Zainab J Alshaikh

**Affiliations:** 1 Hematology, Salmaniya Medical Complex, Manama, BHR; 2 Internal Medicine, Salmaniya Medical Complex, Manama, BHR

**Keywords:** diabetes mellitus, fluid management in dka, complications of dka, diabetic ketoacidosis (dka), diabetes mellitus management, chronic myeloid leukemia (cml), paraneoplastic leukemoid reaction, leukemia

## Abstract

A leukemoid reaction is a rare condition characterized by an elevation in white blood cell count exceeding 50,000 cells/μL in response to severe medical conditions, which can mimic the presentation of chronic myeloid leukemia (CML). Distinguishing between leukemoid reaction and CML depends on a thorough clinical history and comprehensive laboratory evaluation. We present a case of leukemoid reaction associated with severe diabetic ketoacidosis, where the patient's white blood cell count returned to the normal range after the correction of hyperglycemia and electrolyte imbalances.

## Introduction

Leukemoid reaction is a clinical condition characterized by a significant increase in white blood cells ranging from 50,000 to 100,000 per mm^3^, mainly mature neutrophils, and immature granulocytic forms in the peripheral blood (left shift) [1]. It is a rare manifestation of stress or systemic inflammation that has been linked to both malignant and benign disease processes [2]. It is produced by inflammatory reactions or organic stress from a variety of causes, including infections, medications, intoxications, bleeding, and cancer [1]. When defining leukemoid reactions, the actual cut-off value for leukocytes might vary, with lower and higher levels also being used as a cut-off [3].

Diabetic ketoacidosis (DKA) is a hyperglycemic and ketotic condition. Patients frequently complain of nausea, vomiting, and abdominal pain [4]. Infections and treatment non-compliance were the most frequent triggering factors [4]. Diabetic ketoacidosis is distinguished by hyperglycemia greater than 250 mg/dL, a bicarbonate level less than 18 mEq/L, and a pH less than 7.30, as well as ketonemia and ketonuria [5]. Leukemoid reactions are uncommon in cases of simple diabetic ketoacidosis, despite the possibility of modest leukocytosis coinciding with DKA [6]. Our analysis of the literature showed that there are only a few case reports of leukemoid reaction with DKA. Our patient had a significant increase in white blood cells associated with DKA. After conducting extensive tests to rule out hematologic malignancy, the patient was diagnosed with a leukemoid reaction.

## Case presentation

A 23-year-old woman with known type 1 diabetes mellitus, G6PD deficiency, and sickle cell trait presented to the emergency department with dizziness, fatigue, nausea, abdominal pain, and vomiting over the past three days. She was poorly compliant with her diabetes medication (insulin aspart 8 units three times daily and insulin glargine 19 units at bedtime) for two years.

On examination, she was unwell, dehydrated, and pale. She was fully conscious but drowsy. Her vital signs showed a temperature of 36.8°C, blood pressure of 122/80 mmHg, pulse of 90 bpm, and oxygen saturation of 99% on air. Her abdomen was soft, lax, and non-tender, and her spleen was not palpable. No lymph node was palpable all over the body. Other systems were normal.

Urinalysis revealed severe ketonuria and glycosuria. Complete blood count (CBC) showed a low hemoglobin of 3.9 g/dL, platelets of 876 x 10^9/L, and a white blood cell (WBC) count of 75.68 x 10^9/L with a left shift, including 2% myelocytes, 3% metamyelocytes, 6% band forms, 69% segmented neutrophils, 6% monocytes, and 13% lymphocytes. The WBC count was remarkably high, with the appearance of myelocytes and metamyelocytes in the peripheral blood (Table 1). However, her serum C-reactive protein (CRP) level was only 36.1 mg/l while the plasma glucose level was markedly elevated to 450 mg/dl. In addition, the serum total ketone body level was extremely high (14 mmol/l). Arterial blood gas analysis showed severe diabetic ketoacidosis (pH 6.9, bicarbonate (HCO3-) 9 mmol/L, and anion gap 28 mEq/L) (Table 2). The serum exocrine pancreatic enzymes (amylase, lipase, trypsin, and elastase-1) were all within normal levels. Abdominal ultrasound showed only mild hepatomegaly measuring 16.9 cm.

**Table 1:** Hemogram showing the trending of the blood cell count DOA, date of admission; PAD, post-admission day; WBC, white blood cells; RBC, red blood cells; HCT, hematocrit; MCV, mean corpuscular volume; MCH, mean corpuscular hemoglobin; MCHC, mean corpuscular hemoglobin concentration

	DOA	1^st^ PAD	2^nd ^PAD	5^th^ PAD	Normal values
WBC	75.68	48.59	22.6	6.06	3.6-9.9 x 10^9/L
RBC	1.44	2.65	3.12	3.23	3.9-5.2 x 10^12/L
Hemoglobin	3.9	7.2	8.5	9	12.0-14.5 g/dL
HCT	13.90 %	23.6 %	26.7 %	28.6 %	33-45 %
MCV	96.6	89	85.7	88.8	80-97 fL
MCH	26.8	27.4	27.2	28.0	27-33 pg
MCHC	27.7	30.8	31.8	31.6	30-37 g/dL
Platelets	876	743	588	581	150 -400 x 10^9/L
Neutrophils	69 %	72 %	74%	64 %	40 % - 80 %
Lymphocytes	13 %	1 %	19 %	24 %	20 % - 80 %
Monocytes	6 %	1 %	5.8 %	10 %	2 % - 10 %
Eosinophils	-	-	0.1 %	-	1 % - 6 %
Basophils	-	-	0.3 %	-	0.5 % - 1 %
Band forms	6 %	9 %	5 %	1 %	< 1 %
Meta-myelocytes	3 %	9 %	4 %	1 %	-
Myelocytes	2 %	8 %	3 %	-	-
Pro-myelocytes	1 %	-	-	-	-
Blast cells	-	-	-	-	-
Atypical lymphocytes	-	-	-	-	-
Nucleated RBC	50 %	37 %	69 %	170 %	>0.02 x 10/L

**Table 2:** Other laboratory findings DOA, date of admission; DOD, date of discharge; GAD, anti-glutamic acid decarboxylase antibody

	DOA	DOD
Random blood glucose	450 mg/dL	230 mg/dL
Urine analysis	Protein +2 Glucose +3 Ketone bodies +3	-
Sodium	142 mmol/L	140 mmol/L
Potassium	4 mmol/L	3.5 mmol/L
Bicarbonate	9 mmol/L	23 mmol/L
Chloride	105 mmol/L	106 mmol/L
Anion gap	28 mmol/L	11 mmol/L
C-reactive protein	36.18 mg/L	15 mg/L
Serological data	GAD antibody (negative ) Insulin antibody (negative )	-
Microbiological data	Urine culture (sterile) Blood culture (sterile)	-

Peripheral smear showed leukocytosis with a left shift of granulocytes. Complete blood count (CBC) revealed low hemoglobin. Red blood cells (RBCs) showed anisopoikilocytosis, hypochromic microcytic macrocytes, nucleated cells, and teardrop cells. Thrombocytosis was also noted. Clinical correlation was recommended, but the BCR-ABL result was negative.

To treat her diabetic ketoacidosis, an intravenous infusion of saline was given immediately along with a continuous infusion of insulin (6 units/hour) and broad-spectrum antibiotics. Within a few days, her glucose level and metabolic acidosis were controlled. Besides, the WBC count became normal on the fifth day (Table 1).

Laboratory data such as CBC and peripheral blood smear obtained after controlling the plasma glucose level showed normal WBCs (6.9 x 10^9/L) and normal peripheral blood smear cell morphology. Islet-associated antibodies (anti-glutamic acid decarboxylase antibody (GAD) and anti-insulin antibody) were negative (Table 2). Septic workup with urine and blood cultures were negative. Vacuities screening, respiratory profile, and viral profile were also all negative.

Based on these findings, severe diabetic ketoacidosis associated with a leukemoid reaction was diagnosed. She was discharged on insulin therapy and a diet regimen. There has been no recurrence of an abnormal WBC count.

## Discussion

A leukemoid reaction refers to a significant elevation in the number of WBCs (>50,000/μL) within the bloodstream, characterized by pronounced neutrophilia, creating a potential resemblance to leukemia, particularly chronic myeloid leukemia. The initial surge in leukocytes is a result of an accelerated release of cells from the bone marrow, leading to an increased presence of less mature neutrophils in the blood, commonly known as a left shift [7]. It is important to distinguish a leukemoid reaction from chronic myeloid leukemia (CML), as they have distinct clinical presentations and laboratory findings.

A leukemoid reaction is commonly observed in severely ill patients hospitalized with conditions such as sepsis or organ rejection. In contrast, CML typically manifests in outpatient settings, with symptoms like weight loss, sweats, low-grade fever, and splenomegaly. While a palpable spleen is frequently detected in CML patients, it is rare in leukemoid reactions unless accompanied by comorbidities like liver disease [8].

Laboratory findings play a vital role in distinguishing between CML and leukemoid reactions. In CML, the total leukocyte count is usually markedly elevated (median, 100,000/mm³), with counts above 100,000/mm³ being rare, and counts exceeding 150,000/mm³ being almost unheard of in leukemoid reaction. Circulating myelocytes and blasts are more commonly observed in CML but can also be present in both disorders. Platelet number and morphology changes can occur in both conditions, but extreme alterations are more suggestive of CML. RBC changes are not reliable differentiators, except when prominent teardrops are present, indicating a myeloproliferative disorder (MPD). The leukocyte differential is informative, as CML patients typically exhibit absolute basophilia and eosinophilia, whereas infection and excess glucocorticoids result in eosinopenia. The leukocyte alkaline phosphatase (LAP) score, traditionally high in leukemoid reactions and low in CML, has limited utility now due to more sensitive and specific tests for CML (Table 3) [8].

**Table 3:** Differences between leukemoid reactions and CML CML: chronic myeloid leukemia

Characteristic	Leukemoid reaction	CML
Clinical presentation	More common in hospitalized patients with underlying illnesses	Typically diagnosed in outpatient settings
Total leukocyte count	Rarely exceeds 100,000/mm³	Typically extremely high (median, 100,000/mm³)
Absolute basophilia and eosinophilia	Less common	More common
Leukocyte alkaline phosphatase (LAP) score	Traditionally high	Typically low
Management	Treat underlying condition	Treat underlying conditions with tyrosine kinase inhibitors

Here is a summary of the key differences between leukemoid reaction and CML.

Leukemoid reactions are uncommon, and their association with diabetic ketoacidosis is exceptionally rare. The exact pathophysiology underlying this phenomenon remains uncertain. Nonetheless, across multiple studies, heightened leukocyte levels in diabetes patients have been linked to diverse factors, including insulin deficiency, increased inflammatory processes, the secretion of adrenaline and cortisol, and the presence of infection. Furthermore, study findings underscore a robust direct correlation between blood pH and the leukocyte count, signifying that the intensity of blood acidity escalates concomitantly with the number of leukocytes [9].

However, there is a relationship between leukemoid reaction and G6PD deficiency and sickle cell trait because both of these conditions can increase the risk of hemolysis. Hemolysis can release large amounts of inflammatory molecules into the bloodstream, which can trigger a leukemoid reaction [10-11].

Management of leukemoid reactions to DKA primarily revolves around addressing the underlying cause. In this case, aggressive management of DKA itself, early intervention with intravenous fluids, administration of insulin treatment, and correction of electrolyte imbalances are essential to restore metabolic homeostasis and correct the leukemoid response. The improvement of metabolic dysfunction after treatment is gradually resolved, and the patient's leukocytosis subsides.

In general, the occurrence of leukemoid reaction in response to diabetic ketoacidosis (DKA) is infrequent yet poses a significant threat. Timely and effective handling of the fundamental metabolic acidosis is imperative to avert severe complications.

## Conclusions

Leukemoid reactions are rare hematologic conditions characterized by a marked increase in the WBC count. This case highlights the association between leukemoid reactions and diabetic ketoacidosis, emphasizing the importance of considering this entity in the differential diagnosis of leukocytosis in the setting of metabolic derangements. Prompt recognition and appropriate management of leukemoid reactions are vital to prevent unnecessary investigations and potential misdiagnoses.

## References

[1] Sene DR, Watashi DM (2021). Leukemoid reaction in a severe COVID-19 patient. Cureus.

[2] Tarekegn K, Colon Ramos A, Sequeira Gross HG, Yu M, Fulger I (2021). Leukemoid reaction in a patient with severe COVID-19 infection. Cureus.

[3] Achufusi TG, Sharma A, Sapkota B, Mirchia K (2020). Rare case of leukemoid reaction in a patient with severe alcoholic hepatitis. Cureus.

[4] Shahid W, Khan F, Makda A, Kumar V, Memon S, Rizwan A (2020). Diabetic ketoacidosis: clinical characteristics and precipitating factors. Cureus.

[5] (2021). Diabetic ketoacidosis DKA workup. https://emedicine.medscape.com/article/118361-workup.

[6] Burris AS (1986). Leukemoid reaction associated with severe diabetic ketoacidosis. South Med J.

[7] Nola M, Dotlić S (2009). Chapter 9 - the hematopoietic and lymphoid systems. Pathology Secrets (Third Edition).

[8] Hoffman R, Benz EJ Jr., Silberstein LE, Heslop H, Anastasi J, Weitz J (2013). Hematology: Basic Principles and Practice. Hematology: Basic Principles and Practice.

[9] Hosseini SY, Firooz M, Moradi B (2019). Leukocytosis in diabetic ketoacidosis; whether the time has come to start antibiotics? A short literature review. Patient Saf Qual Improve J.

[10] Kato GJ, Piel FB, Reid CD (2018). Sickle cell disease. Nat Rev Dis Primers.

[11] Richardson SR, O'Malley GF (2023). Glucose-6-Phosphate Dehydrogenase Deficiency. https://www.ncbi.nlm.nih.gov/books/NBK470315/.

